# Muscle Conditional Medium Reduces Intramuscular Adipocyte Differentiation and Lipid Accumulation through Regulating Insulin Signaling

**DOI:** 10.3390/ijms18081799

**Published:** 2017-08-18

**Authors:** Haiyin Han, Wei Wei, Weiwei Chu, Kaiqing Liu, Ye Tian, Zaohang Jiang, Jie Chen

**Affiliations:** College of Animal Science and Technology, Nanjing Agricultural University, Nanjing 210095, China; 2014205001@njau.edu.cn (H.H.); wei-wei-4213@njau.edu.cn (W.W.); 2012205001@njau.edu.cn (W.C.); 2015205001@njau.edu.cn (K.L.); 2016205005@njau.edu.cn (Y.T.); 2014105002@njau.edu.cn (Z.J.)

**Keywords:** intramuscular preadipocytes, extracellular micro-environment, insulin receptor, insulin-like growth factor 1 receptor, porcine, adipogenesis

## Abstract

Due to the paracrine effects of skeletal muscle, the lipid metabolism of porcine intramuscular (i.m.) preadipocytes was different from that of subcutaneous (s.c.) preadipocytes. To investigate the development of i.m. preadipocytes in vivo, the s.c. preadipocytes were cultured with muscle conditional cultured medium (MCM) for approximating extracellular micro-environment of the i.m. preadipocytes. Insulin signaling plays a fundamental role in porcine adipocyte differentiation. The expression levels of insulin receptor (*INSR*) and insulin-like growth factor 1 receptor (*IGF-1R*) in i.m. Preadipocytes were higher than that in s.c. preadipocytes. The effects of MCM on adipocyte differentiation, lipid metabolism and insulin signaling transdution were verified. MCM induced the apoptosis of s.c. preadipocytes but not of s.c. adipocytes. Moreover, MCM inhibited adipocyte differentiation at pre-differentiation and early stages of differentiation, while the expression levels of *INSR* and *IGF-1R* were increased. Furthermore, MCM treatment increased adipocyte lipolysis and fatty acid oxidation through induction of genes involved in lipolysis, thermogenesis, and fatty acid oxidation in mitochondria. Consistent with the above, treatment of s.c. adipocytes with MCM upregulated mitochondrial biogenesis. Taken together, MCM can approximate the muscle micro-environment and reduce intramuscular adipocyte differentiation and lipid accumulation via regulating insulin signaling.

## 1. Introduction

The deposition of fat in muscle, recognized by the intramuscular fat (IMF), is an important meat quality. Development of adipocytes located within muscles has been studied. A wide variety of evidence indicated significant differences in both metabolic and secretory functions between intramuscular adipocytes and subcutaneous adipocytes [1,2]. Consequently, intramuscular adipocytes had lower lipogenic enzyme activities, smaller cell sizes, and lower lipid content than subcutaneous adipocytes [3]. However, the mechanism underlying regional differences in adipogenesis still remains unknown.

The differentiation of preadipocytes to mature adipocytes includes activation of adipogenic gene expression and induction of insulin sensitivity [4]. Previous studies reported that insulin is an obligate hormone for preadipocyte differentiation [5,6,7,8]. Insulin signaling is initiated by binding of insulin to the insulin receptor (*INSR*), a receptor tyrosine kinase [9]. During adipocyte differentiation, the number of *INSR* increases approximately 25-fold [10]. Before the appearance of responsiveness of insulin receptor (*INSR*) to insulin during later stages of adipocyte differentiation, insulin mediates its early adipogenic effect via insulin-like growth factor-1 receptors (*IGF-1R*), which is initiated by activation of the tyrosine kinase at the cell surface [10]. Once triggered, the *IGF-1R* phosphorylates insulin receptor substrates-1 (*IRS-1*), a key cytosolic protein substrate, which subsequently activates downstream signal transduction pathways with impact on adipogenic gene transcription [11,12]. Meanwhile, insulin receptor was required for the full differentiation response because of the impaired adipocyte differentiation that was associated with decreased insulin receptor expression [13]. All of these findings suggested the molecular mechanism of insulin action on adipocyte differentiation, but the effects of insulin signaling on porcine intramuscular adipogenesis are not well understand. In this study, we turned our attention to the most proximal steps of insulin signaling for further study, including the *INSR*/*IGF-1R* and the major direct substrate, *IRS-1*.

Intramuscular adipocytes and subcutaneous adipocytes development occurs in the different extracellular micro-environment. Systemic blood circulation provides transport capabilities for endocrine hormones, cytokines (adipokines and myokines) and nutrition for adipose tissue development. However, the development of intramuscular adipocytes are exclusively affected by the paracrine activity of skeletal muscle fibers due to their particular location in close vicinity to muscle fibers. For example, myokines interleukin-15 (IL-15), myostatin (MSTN) and irisin inhibit adipocyte differentiation and fat deposition via a skeletal muscle to fat signaling pathway [14,15,16]. In these in vitro studies, it just verified the role of one purified cytokine present in the micro-environment. It is possible to approximate the tissue micro-environment using commercially available products. However, in all cases, it is unlikely that the material used represents the specific combination of factors that exist within the tissue micro-environment. To address these challenges, we extracted the interstitial fluid from skeletal muscle, which can be applied as muscle conditional cultured medium (MCM) for approximating the extracellular micro-environment of i.m. preadipocytes in vivo. The subcutaneous preadipocytes were cultured in the MCM to study intramuscular adipose tissue development, including adipocyte differentiation and adipose tissue expansion.

In the present study, the mechanism of insulin signaling in porcine adipocyte differentiation was verified. Meanwhile, the effects of extracellular micro-environment on adipocyte development were detected using skeletal muscle interstitial fluid that was applied as MCM. Furthermore, the molecular mechanism of MCM action on adipogenesis by modulating insulin signal transduction was investigated. The goal of this study was to understand the intramuscular adipogenesis with emphasis on cell micro-environment interactions that are pivotal in regulating adipocyte formation.

## 2. Results

### 2.1. Cellular Differentiation and Identification of Porcine Intramuscular and Subcutaneous Preadipocytes

**Figure 1:**
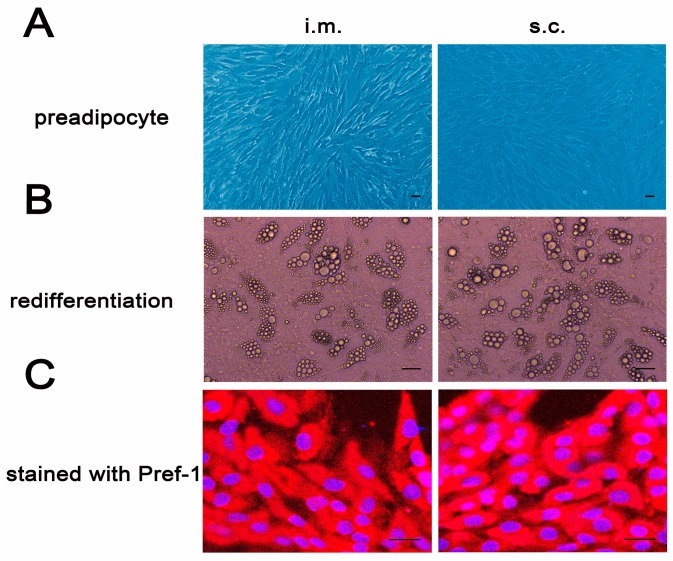
Identification of porcine i.m. and s.c. preadipocytes. The dedifferentiated porcine intramuscular (i.m.) and subcutaneous (s.c.) preadipocytes showed fibroblast-like morphology (**A**); The i.m. and s.c. preadipocytes were induced in adipogenic medium at confluence. Nine days later, the i.m. and s.c. preadipocytes were re-differentiated to mature adipocytes with lipid droplets (**B**); The isolated i.m. and s.c. preadipocytes were seeded in 12-well plates, and the cells of day 3 were detected by Pref-1 immunofluorescent staining (**C**). Scale bar = 100 μm.

The intramuscular (i.m.) and subcutaneous (s.c.) preadipocytes which isolated using the ceiling culture method were resuspended in culture medium with an irregular triangular appearance when freshly inoculated. After three days of culture in growth medium, both i.m. and s.c. preadipocytes showed fibroblast-like morphology (Figure 1A). Both of them could redifferentiate into mature adipocytes with lipid droplets following by adipogenic stimulation at confluence (Figure 1B). Pref-1 is an adipocyte-specific protein, with a special expression in preadipocytes, inhibiting adipose differentiation in the early differentiation stage [17,18]. The fibroblast-like cells were identified by immunofluorescence staining of Pref-1. As shown in Figure 1C, positive Pref-1 reactions demonstrated that the isolated cells were preadipocytes with high purity. In conclusion, the date above confirmed that the fibroblast-like cells were porcine i.m. and s.c. preadipocytes, respectively.

### 2.2. Inhibition Differentiation of Porcine Adipocyte by Downregulating the Expression of IGF-1R or INSR

Insulin initiates its pleiotropic effects on cellular growth and metabolism by binding to its specific cell-surface receptor, *INSR* and *IGF-1R*. To elucidate the function of *INSR* and *IGF-1R* in determining adipocyte differentiation, s.c. preadipocytes were transfected with *INSR* and *IGF-1R* siRNAs, respectively. After 24 h, the s.c. preadipocytes were induced to differentiate following adipogenic stimulation.

**Figure 2:**
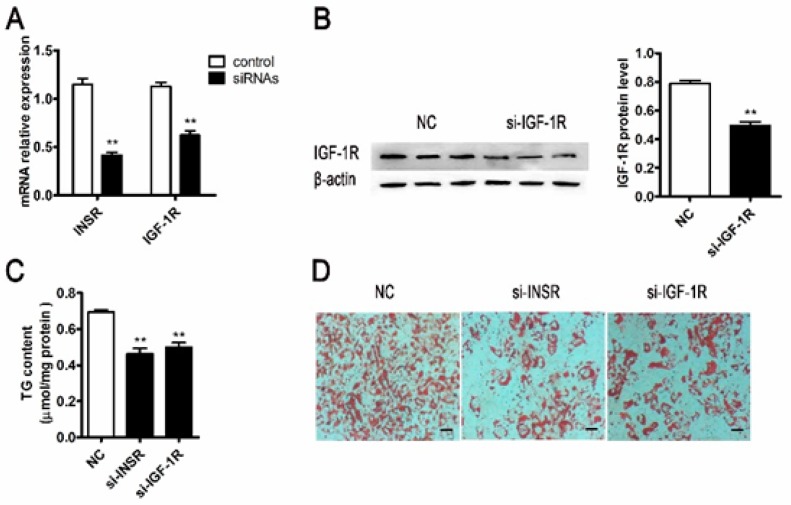
Downregulation of insulin receptor (*INSR*) or IGF-1 receptor (*IGF-1R*) by siRNA interference decreased porcine adipocyte differentiation. The siRNAs targeting INSR and IGF-1R (si-INSR, si-IGF-1R) were transfected into porcine s.c. preadipocytes. The *INSR*/*IGF-1R* mRNA (**A**) (*n* = 6) and protein levels (**B**) (*n* = 3) were verified after transfected with siNC and si-INSR/si-IGF-1R at 24 and 48 h, respectively; Transfected s.c. preadipocytes were induced to differentiate for nine days; The intracellular accumulation was determined by cytoplasm triglyceride content (**C**) (*n* = 4) and Oil Red O staining (**D**) (*n* = 3). The mRNA expression differences were normalized to Ribosomal protein lateral stalk subunit P0 (*RPLP0*) mRNA level. The β-actin bands served as an internal control for protein loading. ** *p* < 0.01, Scale bar = 200 μm.

Knockdown efficiency was examined by qPCR and immunoblotting for *INSR* and *IGF-1R*. The mRNA expression levels of *INSR* and *IGF-1R* were significantly reduced by 64% and 45% in siRNA transfected cells, respectively (Figure 2A). The protein expression level of *IGF-1R* was also reduced significantly (Figure 2B). However, the protein expression of *INSR* could not be examined due to the pig INSR antibody was not available.

Knockdown of *INSR* using siRNA decreased adipocyte differentiation showing by lower cytoplasm triglycerides content and the lipid droplets in mature adipocytes compared with the control (NC) (Figure 2C,D). Similar results were obtained from *IGF-1R* knockdown (Figure 2C,D). These results indicated that siRNA interference not only suppressed the expression of *INSR* and *IGF-1R* but also blocked hormone-induced adipocyte differentiation.

### 2.3. The Differences of Insulin Signaling between Porcine i.m. Preadipocytes and s.c. Preadipocytes

**Figure 3:**
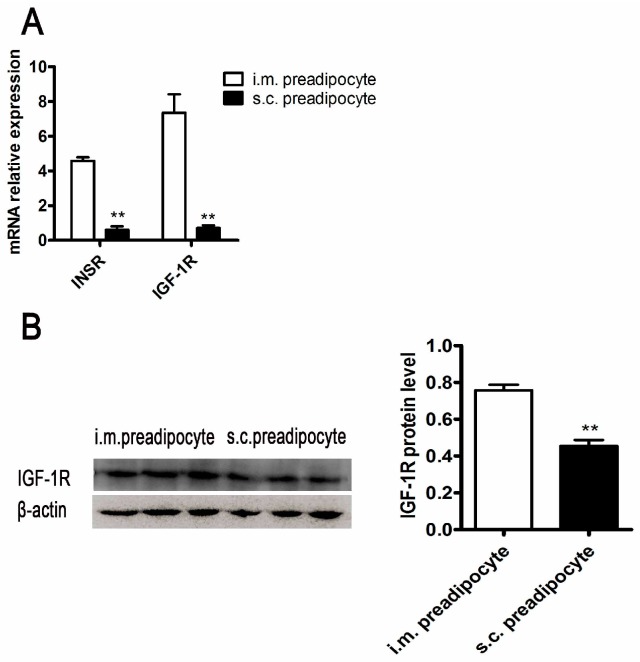
*INSR* and *IGF-1R* expression pattern in i.m. and s.c. preadipocytes. The comparison of *INSR* and *IGF-1R* mRNA expression levels in i.m. and s.c. preadipocytes (**A**) (*n* = 3); The protein expression level of *IGF-1R* in i.m. and s.c. preadipocytes was detected by Western blot and subsequently quantified. (**B**) (*n* = 3). The mRNA expression differences were normalized to *RPLP0* mRNA level. The β-actin bands served as an internal control for protein loading. ** *p* < 0.01.

The expression levels of *INSR* and *IGF-1R* between i.m. and s.c. preadipcytes were compared to analyze the regional differences in adipogenesi. The mRNA expression levels of *INSR* and *IGF-1R* in i.m. preadipocytes were both significantly higher than those in s.c. preadipocytes (Figure 3A). Accordingly, the protein expression pattern of *IGF-1R* between i.m. and s.c. preadipocytes was consistent with the mRNA (Figure 3B). However, the protein expression of *INSR* could not be examined due to the pig *INSR* antibody was not available.

### 2.4. MCM Identification and Its Effect on Adipocyte Differentiation

**Figure 4:**
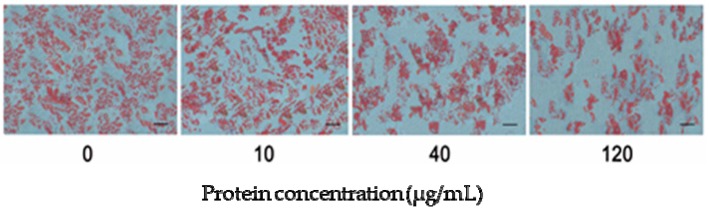
The inhibitory effect of muscle conditional medium (MCM) on s.c. preadipocytes differentiation. Confluenced s.c. preadipocytes were incubated in adipogenic medium supplemented with an increasing amount of MCM at the indicated total protein concentration (*n* = 3). After nine days, adipocytes were staining with Oil Red O. Scale bar = 200 μm.

The protein factors in the muscle conditional medium (MCM) were identified by label free quantitation (date not shown). There were so many kinds of proteins, including cytokines released from muscle tissue, adipose tissue and other endocrine organ. The s.c. preadipocytes were cultured with MCM at different concentrations to verify the effect of MCM on porcine adipocyte differentiation. During the differentiation, the s.c. preadipocytes were simply incubated in adipogenic medium supplemented with MCM at 0, 10, 40, and 120 μg·mL^−1^ total protein concentration. Nine days later, intracellular oil droplets were stained with Oil Red O. As indicated in Figure 4, MCM suppressed lipid accumulation with significant effects at 120 μg·mL^−1^ total protein concentration. The inhibition effect of MCM on adipocyte differentiation was consistent with the development of i.m. preadipocytes in vivo. The amount of MCM at 120 μg·mL^−1^ was used for further study.

### 2.5. MCM Induced Apoptosis of Porcine Preadipocytes

**Figure 5:**
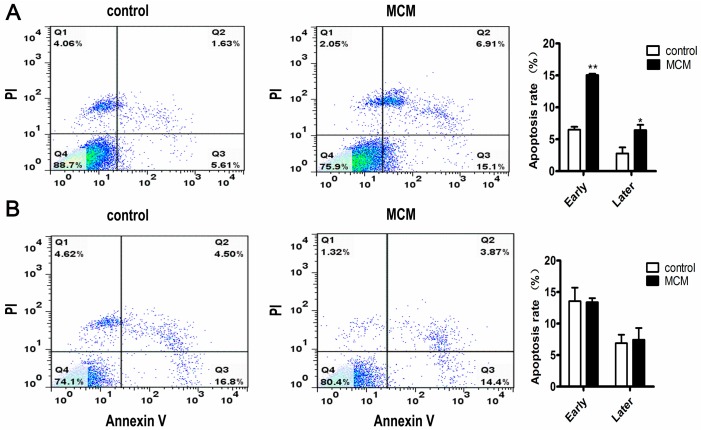
Flow cytometric detection and quantification of MCM-induced apoptosis in porcine s.c. preadipocytes and s.c. adipocytes. Porcine s.c. preadipocytes (**A**) and s.c. adipocytes (**B**) were cultured for three days with MCM versus physiological saline, and stained with fluorescein isothiocyanate (FITC)-labled annexin V (Annexin V-FITC) and propidium iodine (PI); then, the cell apoptosis was analyzed. *n* = 3, * *p* < 0.05, ** *p* < 0.01, Scale bar = 200 μm.

Porcine s.c. preadipocytes and s.c. adipocytes were respectively treated with MCM at 120 μg·mL^−1^ total protein concentration for three days and then stained with fluorescein isothiocyanate (FITC)-labled annexin V (Annexin V-FITC and propidium iodine (PI) to detect cell apoptosis. Flow cytometric detection indicated that MCM induced significantly apoptosis in s.c. preadipocytes when compared with the control group (Figure 5A). The early and late apoptosis percentage was observed from 6.5% to 15.1% (*p* < 0.01), 2.8% to 6.4% (*p* < 0.05), respectively (Figure 5A). However, the apoptosis effects of MCM on s.c. adipocytes differed from that on s.c. preadipocytes. There was no difference in the apoptosis rate between control and the MCM-treated group (Figure 5B).

### 2.6. MCM Suppresses Adipocyte Differentiation during the Pre-Differentiation to the Middle Stage

To elucidate the effective duration of activity of MCM during differentiation, s.c. preadipocytes were treated for three days with MCM at 120 μg·mL^−1^ total protein concentration for four different time intervals: pre-differentiation (day −3 to 0), early (day 0 to 3), middle (day 4 to 6), and late (day 7 to 9) stages of differentiation.

**Figure 6:**
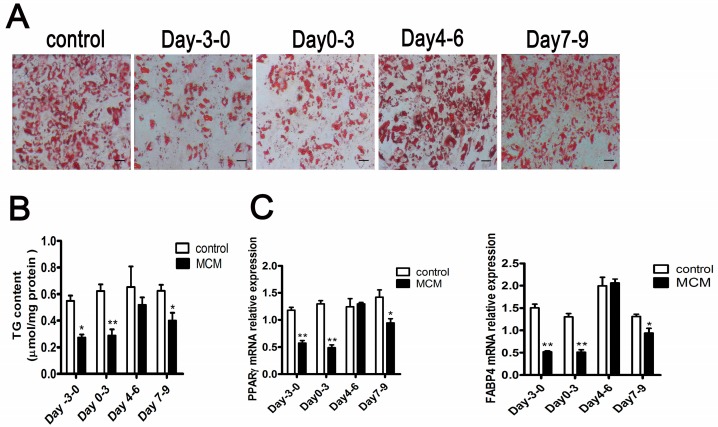
The effective duration of activity of MCM during adipocyte differentiation. s.c. preadipocytes were treated for three days with MCM at 120 μg mL^−1^ total protein concentration for four different time intervals: pre-differentiation (day –3 to 0), early (day 0 to 3), middle (day 4 to 6), and late (day 7 to 9) stages of differentiation. After nine days of differentiation, the effects of MCM on intracellular oil droplets (**A**) (*n* = 3), triglycerides content (**B**) (*n* = 3) and peroxisome proliferator-activated receptor γ (*PPARγ*) and fatty acid binding protein (*FABP4*) mRNA expression (**C**) (*n* = 6) were verified. The mRNA expression differences were normalized to *RPLP0* mRNA levels. Triglyceride content was normalized to protein content. * *p* < 0.05, ** *p* < 0.01, Scale bar = 200 μm.

The effect of MCM on adipocyte differentiation was different for the different stages. MCM significantly suppressed lipid accumulation with treatment at the pre-differentiation and early stage of differentiation, respectively, as indicated by less lipid droplets, decreased triglyceride content, and lower expression of adipocyte makers, peroxisome proliferator-activated receptor γ (*PPARγ*) and fatty acid binding protein (*FABP4*) (Figure 6A–C). While there was no difference in adipocyte differentiation with treatment at the middle stage of differentiation (Figure 6A–C). When treated at the late stage of differentiation, MCM decreased triglyceride content and lower expression of adipocyte makers, *PPARγ* and *FABP4* (Figure 6B,C). However, there was no difference in stained lipid droplets evaluated by Oil Red O staining (Figure 6A). These observations suggest that MCM inhibits adipocyte differentiation during pre-differentiation to middle stages of differentiation. Moreover, MCM reduces adipose tissue expansion by inhibiting lipid accumulation in adipocyte.

### 2.7. MCM Increases INSR and IGF-1R Expression and Activates Insulin Signaling

During the process of adipocyte differentiation treatment with MCM, we focused on *INSR*/*IGF-1R*-*IRS-1* expression in the insulin signaling cascade by qPCR and immunoblotting.

**Figure 7:**
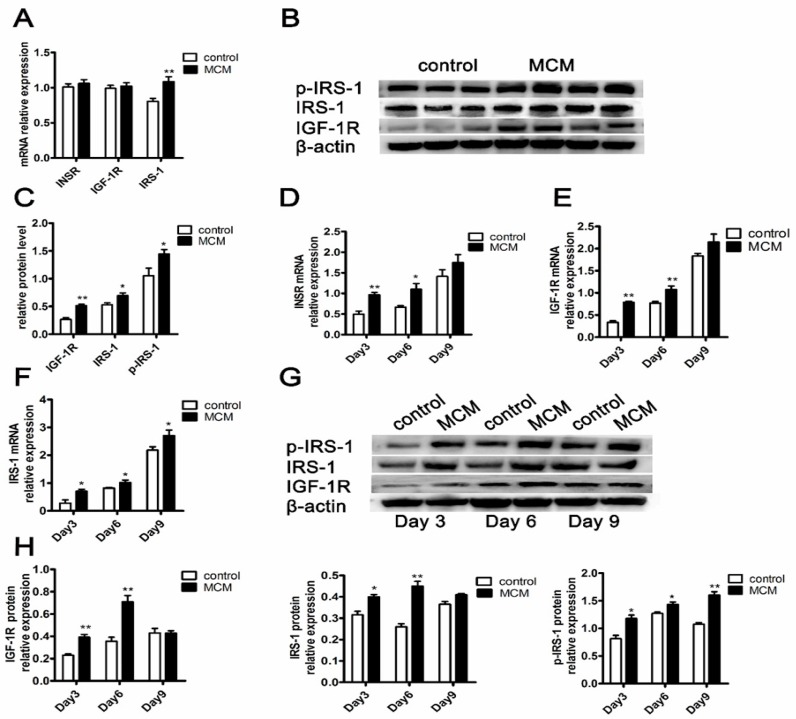
The genes expression patterns in the *INSR*/*IGF-1R*-*IRS-1* signaling. The mRNA expression of *INSR*, *IGF-1R* and *IRS-1* with MCM treatment at the pre-differentiation stage of adipocyte differentiation for three days (**A**) (*n* = 4); The protein abundance of *IGF-1R*, *IRS-1*, and *p-IRS-1* with MCM treatment at pre-differentiation stage for three days (**B**) and protein quantified was shown (**C**) (*n* = 3 for control, *n* = 4 for treatment); The mRNA expression of *INSR* (**D**) (*n* = 6), *IGF-1R* ((**E**) *n* = 6), and *IRS-1* ((**F**) *n* = 6) with MCM treatment for different durations; The protein abundance of *IGF-1R*, *IRS-1*, and *p-IRS-1* with MCM treatment during different differentiation durations (**G**) and protein quantified was shown ((**H**) *n* = 3); The mRNA expression differences were normalized to *RPLP0* mRNA level. The β-actin bands served as an internal control for protein loading, *p-IRS-1* (Try) expression was normalized to *IRS-1* expression. * *p* < 0.05, ** *p* < 0.01.

In the pre-differentiation stage of differentiation, MCM only led to a marked increase in *IRS-1* mRNA expression (*p* < 0.01) (Figure 7A), while significantly increase in the protein expression of *IGF-1R*, *IRS-1* and also the phosphorylation level of *IRS-1*(Try) (*p* < 0.01) (Figure 7B,C). During the different durations of differentiation, there was an increase in both the mRNA (Figure 7D–F) and protein expression (Figure 7G,H) of *IGF-1R*, *IRS-1* and also the phosphorylation level of *IRS-1* (Try) (Figure 7G,H) in MCM treated cells, with a significant increase mainly at the early and middle stages of differentiation. These data demonstrated that MCM increases *INSR* and *IGF-1R* expression and activates insulin signaling.

### 2.8. MCM Increases Adipocyte Lipolysis and Fatty Acid Oxidation

**Figure 8:**
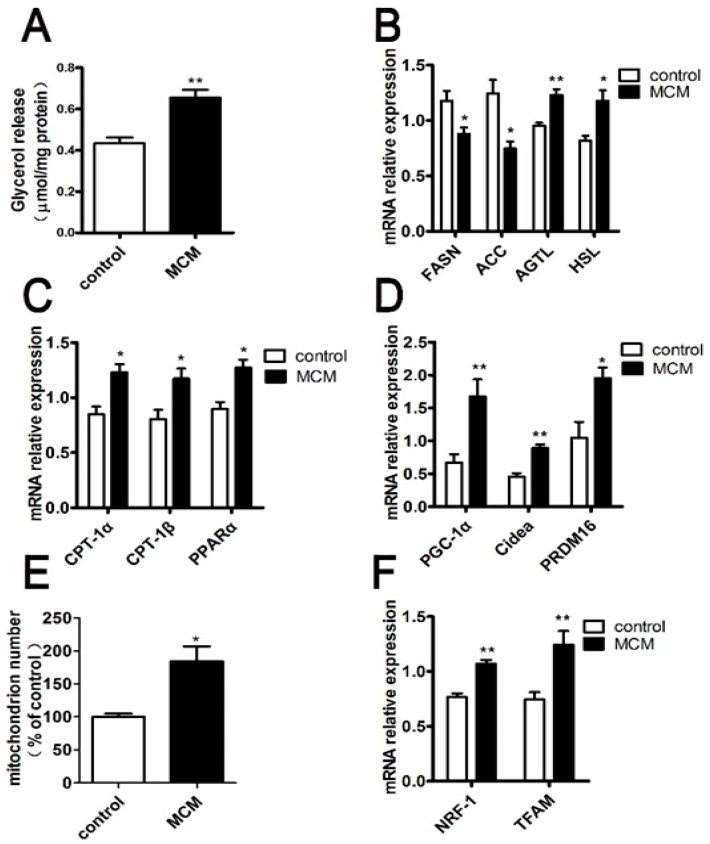
Effect of MCM on adipocyte lipolysis and fatty acid oxidation. Fully differentiated adipocytes were incubated with the Dulbecco’s Modified Eagle’s medium (DMEM) (no phenol red) with MCM for 24 h to verify the lipolytic effects of MCM. The glycerol levels in the culture medium were assayed (**A**); Adipocytes were cultured with MCM for three days. The expression levels of genes involved in lipolysis (*ATGL* and *HSL*) and fatty acid synthesis (*FASN* and *ACC*) (**B**) were detected by q PCR, respectively; Relative mRNA expression of genes responsible for fatty acid oxidation (*PPARα*, *Cpt-1α*, and *Cpt-1β*) (**C**) and thermogenesis (*PGC-1α*, *PRDM16*, and *Cidea*) (**D**) was detected; Relative abundance of mitochondrial DNA (mtDNA) (**E**) and the genes responsible for mitochondrial biogenesis (*PGC-1α*, *TRFM*, and *NRF*) (**F**) were detected. Dates were normalized to the mRNA levels of *RPLP0*. *n* = 6, * *p* < 0.05, ** *p* < 0.01.

The differences of lipid metabolism between control and MCM-treated s.c. adipocytes were analyzed by determining the content of glycerol release and the expression patterns of the genes involved in lipid metabolism. The lipolytic effects of MCM on porcine adipocyte were determined by treating s.c. adipocytes (day 9 after differentiation medium stimulation) with MCM for 24 h. As shown in Figure 8A, MCM induced greater release of glycerol into the culture medium than control did. Additionally, MCM was added to the cells at day 6 of s.c. adipocyte differentiation for a total of three days of stimulation. Quantitative real-time PCR (qPCR) confirmed the reduction expression of genes encoding enzymes involved in fatty acid synthesis, including fatty acid synthase (*FASN*) and acetyl CoA carboxylase (*ACC*) (Figure 8B). Consistent with the increase of lipolysis, the mRNA expression of adipose triglyceride lipase (*ATGL*) and hormone sensitive lipase (*HSL*), which are the major lipases related to lipolysis, significantly increased in MCM-treated adipocytes (Figure 8B).

The expression of genes involves in fatty acid oxidation, including carnitine palmitoyl transferase 1α (*Cpt1α*), carnitine palmitoyl transferase 1β (*Cpt1β*) and peroxisome proliferator activated receptor α (*PPARα*), was also enhanced in MCM-treated adipocytes (Figure 8C). Moreover, treatment of differentiated adipocytes with MCM resulted in a robust increase of peroxisome proliferator activated receptor-1α (*PGC-1α*) mRNA levels together with an upregulation of the expression of additional key thermogenesis genes such as PR domain containing 16 (*PRDM16*) and cell death-inducing DFFA-like effector A (*Cidea*) (Figure 8D). Furthermore, the effects of MCM on mitochondrial biogenesis were also evaluated. Importantly, MCM induced a robust increase in mitochondrial content (Figure 8E). Compared to the control group (100%), the mitochondrial DNA copy number in MCM-treated groups increased significantly (184.5 ± 22.5%, *p* < 0.05) (Figure 8E). The mRNA levels of genes implicated on mitochondrial biogenesis, including *PGC-1α*, nuclear respiratory factor-1 (*NRF-1*), mitochondrial transcription factor A (*TFAM*) were higher in MCM-treated adipocytes (Figure 8D–F). Taken together, the date suggested that MCM improved lipid metabolism by increasing adipocyte lipolysis and fat acid oxidation.

## 3. Discussion

Regional variation in fat distribution affects economic values in pork production. Larger amounts of intramuscular fat can improve pork quality [19], whereas subcutaneous fat is considered to be one of the main sources of waste. The lipid metabolism and differentiation levels of i.m. adipocytes differed from that of s.c. adipocytes [3,20,21]. To study the molecular mechanism difference between i.m adipocytes development and s.c. adipocytes development, the i.m. and s.c. preadipocytes were isolated by the ceiling culture technique (Figure 1). These i.m. and s.c. preadipocytes originate from a homogeneous cell population derived from a single fraction of mature adipocytes. The cells proliferate extensively until they become confluent and differentiate into mature adipocytes upon treatment with insulin, dexamethasone (Dex) and 3-isobutyl-1-methylxanthine (IBMX) (Figure 1B). The cells proliferate more effectively compared to the stromal-vascular fraction isolated from traditionally collagenase technique [22].

The variety biology effects of insulin in adipocytes are mediated by insulin receptor (*INSR*) as well as IGF-1 receptor (*IGF-1R*), both of them belong to the family of receptor tyrosine kinases [23]. Importantly, the two receptors activate common intracellular pathways by using similar mechanisms [24]. However, one receptor cannot functionally compensate when the other one is absent [25]. Thus, the adipogenesis role of *INSR* and *IGF-1R* were verified in this study. Downregulation of *INSR* and *IGF-1R*, respectively, lead to the reduction of adipocyte differentiation (Figure 2), which consisted of previous results in mice adipogenesis research [13,26,27]. In vivo study, mice with the fat-specific insulin receptors and/or IGF-1 receptors knockout have reduced fat mass [26,27]. Moreover, decrease in expression of 3T3-L1 preadipocyte insulin receptor impaired adipocyte differentiation [13]. All of these findings demonstrated that *INSR* and *IGF-1R* signaling are essential molecules for adipogenesis.

The regional differences of *INSR* and *IGF-1R* expression were compared between i.m. preadipocyte and s.c. preadipocyte. The mRNA expression level of *INSR* and *IGF-1R* in i.m. preadipocytes was higher than that in s.c. preadipocytes (Figure 3A). The similar gene expression pattern was seen in protein level (Figure 3B). Compared with subcutaneous adipose tissue, the higher expression level of *INSR* and *IGF-1R* in intramuscular adipose tissue did not render more fat in this regional, suggesting that the extracellular micro-environment of the i.m. preadipocytes has an important influence.

Adipose tissue is a very important organ for secretion of many endocrine and paracrine factors, collectively referred to adipokines [28,29]. Accordingly, the skeletal muscle has also been identified as an endocrine organ that produces and releases cytokines, which were named “myokines” [30,31,32]. These cytokines influence adipose tissue development in coordination with endocrine hormones via endocrine, paracrine, and autocrine signals [28,33,34]. Due to the particular location in close vicinity with muscle fibers, the myokines have more influence on i.m. adipocytes metabolism than s.c. adipocytes. To approximate the effects of extracellular micro-environment on i.m. preadipocyte development in vivo, the s.c. preadipocytes were cultured with MCM. In contrast to one or more purified hormones or cytokines, MCM represents the specific combination of factors that exist within the tissue micro-environment.

During the process of adipocyte differentiation, lipid accumulation reduced significantly when s.c. preadipocytes were treated with MCM at pre-differentiation and early stages of differentiation, while the expression levels of *INSR* and *IGF-1R* were increased (Figure 6 and Figure 7). The suppression effects of MCM on adipgenesis were supported by previous studies that myocytes suppressed adipocyte differentiation in pig and mouse research [35,36]. Similarly, the accumulation of *INSR* and *IGF-1R*, resulting from MCM treatment, did not render cells more adipogenic. The impaired differentiation was partially due to the induction apoptosis effects of MCM on porcine s.c. preadipocytes (Figure 5). Many myokines, such as interleukin-6, induced lipolysis and fatty acid oxidation in adipocytes [30]. The present study revealed that MCM induced adipocytes lipolysis and fatty acid oxidation in mitochondria (Figure 8). Importantly, the expression levels of genes involved in lipolysis and fatty acid oxidation were increased, while fatty acid synthesis related genes, including *FASN* and *ACC*, were decreased. These date may relate to the reduction of triglyceride content in adipocytes when treated s.c. adipocytes with MCM at the late stage of differentiation (Figure 6B,C).

To further verify the fatty acid oxidation in mitochondria, we evaluated the effects of MCM on mitochondrial biogenesis. Consistent with fatty acid oxidation, the mitochondrial DNA copy number and expression levels of marker genes responsible for mitochondrial biogenesis were increased significantly (Figure 8E,F). The date indicated that MCM could upregulate the mitochondrial biogenesis in s.c. adipocytes.

There are two different types of adipose tissue, namely white adipose tissue (WAT) and brown adipose tissue (BAT). The main function of WAT is to store energy, whereas the BAT is specialized to dissipate energy as heat. Generally, brown adipocytes are predominantly located in BAT. It can also emerge among WAT through the browning process. Cold exposure, hormones, enzymes, transcription factors, and microRNAs have recently been shown to drive the so-called browning process [37,38]. *PGC-1α* and *PRDM16* were required for the switch from WAT to beige cells [39]. Moreover, *PGC-1α* and *Cidea* were usually regarded as the brown adipogenic marker in the regulation of brown and beige fat [40,41]. In the current study, MCM indeed increased the major thermogenesis genes expression, concluding *PGC-1α*, *Cidea* and *PRDM16*, in s.c. adipocytes (Figure 8D). Furthermore, Figure 8C,E revealed a significantly higher fatty acid oxidation capacity and mitochondrial copy number in MCM-treated s.c. adipocytes than that in control cells. These findings indicated that MCM might promote the white to brite trans-differentiation.

## 4. Materials and Methods

### 4.1. Porcine Preadipocytes Isolation, Culture and Identification

All the animals used in this experiments were performed according to “The Guidelines for the Care of Laboratory Animals” enacted by the Ministry of Science and Technology of People’s Republic of China. Three-days-old Suhuai pigs from Animal Husbandry Group in Chang Shu of China were killed via intraperitoneal injection of pentobarbital sodium (50 mg/kg body weight) followed by exsanguinations. Dedifferentiated intramuscular (i.m.) and subcutaneous (s.c.) preadipocytes were isolated using the ceiling culture method. Briefly longissimus dorsi muscle tissue and subcutaneous adipose tissue were sampled, respectively, and cut into pieces followed by digested with 1 mg/mL type I collagenase for about 2 h at 37 °C in a shaking water bath. Then, the two kinds of digested tissues were filtered with 75-μm steel mesh, and cells were washed twice with DMEM by centrifugation at 200× *g* for 10 min. The mature adipocytes were collected from the floating layer and placed into T25-cm^2^ flasks completely filled with DMEM containing 10% fetal bovine serum (FBS). Flasks containing mature adipocytes were inverted and then incubated in a 5% CO_2_ incubator at 37 °C for about 10 days. Mature adipocytes were able to dedifferentiate into proliferative-competent preadipocytes. The excess cell culture media was removed and the culture flask was turned upside-down. The medium was changed every 2 days.

The isolated preadipocytes were cultured in Dulbecco’s Modified Eagle’s medium (DMEM) containing 10% FBS and 1% penicillin-streptomycin at 37 °C in 5% CO_2_. When reaching confluence, the preadipocytes were induced to differentiate into adipocytes by culturing in differentiation medium containing 10% FBS-DMEM supplemented with dexamethasone (0.25 mM), IBMX (1 mM), and insulin (5 μg mL^−1^) (DMI) (Sigma-Aldrich, Shanghai, China). After 3 days, the medium was replaced with 10% FBS-DMEM until day 9. Fresh new medium was changed every 2 days.

The i.m. and s.c. preadipocytes were seeded in 12-well plates to demonstrate that the isolated cells were preadipocytes with high purity. Briefly, the two types of cells were fixed in ice-cold 4% paraformaldehyde for about 10 min, then permeabilized with 1% Triton X-100 for 10 min, and incubated with 5% bovine serum albumin (BSA) for 1 h at room temperature. After washing 3 times with PBS later (5 min each), the cells were incubated with the primary antibodies of rabbit anti-porcine Pref-1 (DLK) (Santa Cruz Biotech Inc., Santa Cruz, CA, USA; sc-25437, 1:100 dilution) overnight at 4 °C. The cells were then washed twice with PBS and incubated with the secondary antibody of kFluor594 labeled goat anti-rabbit IgG antibodies (Keygen Biotech, Nanjing, China; kgif008, 1:500 dilution) for 1 h at room temperature in the dark. The verified images were obtained using a Confocal Laser Scanning Microscope (Carl Zeiss LSM700, Oberkochen, Germany).

### 4.2. Oil Red O Staining and Triglyceride Measurements

To quantify lipid accumulation, Oil Red O staining and cytoplasm triglyceride content were performed on day 9 of adipocyte differentiation. Briefly, the adipocytes were washed twice with phosphate-buffered saline (PBS), fixed with 10% phosphate-buffered formalin for 15 min, stained with Oil Red O working solution for 30 min at room temperature, and then washed for 20 s with 60% isopropanol to remove the excess stain. The stained lipid droplets within adipocytes were visualized by Leica Inverted Fluorescence Microscope (Wetzlar, Germany).

Measurement of intracellular triglyceride levels were performed by Triglyceride assay kit (Applygen Technologies Inc., Beijing, China) according to the manufacturer’s instructions. Triglyceride levels were normalized to protein concentration. Protein content was measured by a BCA Protein Assay Kit (Beyotime, Shanghai, China) according to the manufacturer’s instructions.

### 4.3. mRNA Expression Analysis

The total RNA from cells was extracted with Trizol reagent (Invitrogen, Carlsbad, CA, USA) according to the manufacturer’s instructions. The mRNA expression levels of related genes were detected by Synergy brands (SYBR) Green Dye quantitative real-time PCR (qPCR) (Takara, Dalian, China) with the Applied Biosystems StepOneTM detection System (Applied Biosystems, Foster City, CA, USA). Samples were run in triplicate for each experiment and were normalized to Ribosomal protein lateral stalk subunit P0 (*RPLP0*) to determine relative expression level. Primer sets used in this study were provided in Table 1.

### 4.4. Isolation and Identification of Interstitial Fluid from Skeletal Muscle by a Centrifugation Method

Longissimus dorsi muscle (~20 g) were excised, flushed with saline to remove the possible blood and intracellular fluid from the surface, blotted gently with tissue paper to remove excess saline, and transferred to 50 mL centrifuge TUBES used for isolation of muscle interstitial fluid [42]. To reduce the risk of muscle cell compression, the centrifugation speed was 1000× *g* for 10min. The interstitial fluid accumulated at the bottom of tube was collected, filtered through a 0.2 μm filter, analyzed for protein content, stored at −80 °C and designed as muscle conditioned medium (MCM). In this study, the overall process was carried out at 4 °C. The protein factors in the muscle conditional medium (MCM) were identified by label free quantitation.

### 4.5. siRNAs Trasfection

Porcine s.c. preadipocytes were transfected with siRNA oligos using lipofectamine 2000 transfection reagent (Invitrogen, Carlsbad, CA, USA) following the manufacturer’s manual. The cell were 80% confluence during transfection. The siRNA/lipofectamine 2000 complexes were prepared in opti-MEM media and added dropwise to the cells.

### 4.6. Western Blotting Analyses

The cellular total protein was extracted via addition of RIPA lysis buffer (Beyotime, Shanghai, China) to the cell pellets on ice for 30 min, and protein concentration was measured with a BCA protein quantification kit (Beyotime, Shanghai, China). The protein samples (50 μg) were separated by sodium dodecyl sulphate-polyacrylamide gel electrophoresis (SDS-PAGE), transferred to polyvinylidene fluoride membranes (PVDF) and blotted with specific primary antibodies for IGF-1R (Santa Cruz Biotech Inc., sc-713, 1:200 dilution), IRS-1 (Santa Cruz Biotech Inc, sc-560, 1:200 dilution), p-IRS-1 (Tyr 632) (Santa Cruz Biotech Inc., sc-17196, 1:200 dilution ), and β-actin (Santa Cruz Biotech Inc., sc-47778, 1:1000 dilution). After incubation with horseradish-peroxidase-conjugated (HPR) secondary antibody for 2 h at room temperature, the protein expression was visualized with an enhanced chemiluminescent reagent (ECL, Thermo Fisher Scientific, City, MA, USA) by Versa DosTM 4000 MP (Bio-Rad Laboratories, Munich, Germany).

### 4.7. Glycerol Release

The s.c. adipocytes (day 9 after differentiation medium stimulation) were washed once with PBS gently and then incubated with DMEM (no phenol red and FBS) (Wisent Biotechnology, Nanjing, China) containing with or without MCM for 24 h. The culture medium of samples were collected and glycerol levels in the media were assayed using a glycerol kit (Applygen Technologies Inc., Beijing, China) according to the manufacturer’ instructions.

### 4.8. Analysis of Mitochondrial DNA Content

The mitochondrial DNA (mtDNA) content was assessed using a modification of the quantitative real-time PCR (qPCR) as described previously [43,44]. Briefly, the total DNA was extracted from s.c. adipocytes using DNA extraction kit (Jiancheng Bioengineering Institute, Nanjing, China; d1700-50). The relative amount of mtDNA was quantified by comparison of a mitochondrial target, the cytochrome c oxidase subunit II (*COX2*) with a nuclear target, ribosomal protein lateral stalk subunit P0 (*RPLP0*). Quantitative real-time PCR was performed using Applied Biosystems StepOneTM detection System (Applied Biosystems, Foster City, CA, USA). For quantification, reference curves that were serial dilutions of a standard DNA were used. Primer sequences of *COX2* and *RPLP0* for qPCR was 5′-AGGACGACTAAACCAAACA-3′, 5′-AATGGGACAAGTTCAAGTA-3′ and 5′-ATGGCAGCATCTACAACC-C-3′, 5′-AGACAAAGCCAGGACCCAC-3′, respectively. A ratio between *COX2* and *RPLP0* was calculated (*COX2*/*RPLP0*), which was used as the mtDNA content in the present study. Each sample was measured at least in triplicate, and mean values were calculated.

### 4.9. Ethics Statement

All experiments were performed in accordance with the guidelines of the regional Animal Ethics Committee and were approved by the Institutional Animal Care and Use Committee of Nanjing Agricultural University (NJAU-CAST-2014-179, 1 January 1978).

### 4.10. Statistical Analysis

Results are expressed as the mean ± SEM. Graphpad Prism software (Graphpad Prism 5 V 5.01, GraphPad Prism Software Inc., San Diego, CA, USA) was used for all analyses. The comparison performed using Student’s *t*-test or one-way NAOVA. Minimal level of significance was set at *p* < 0.05. All experiments were performed a minimum of three times.

## 5. Conclusions

INSR and IGF-1R signaling are essential molecules for porcine adipogenesis. MCM induced an apoptotic effect on preadipocytes. Moreover, MCM inhibited adipocyte differentiation at pre-differentiation and early stages of differentiation, while the expression levels of INSR and IGF-1R were increased. The lipid metabolism was improved via induction of adipocytes lipolysis, fatty acid oxidation and expression levels of key thermogenesis genes by MCM. In conclusion, the effects of MCM on lipid metabolism could approximate the development of i.m. preadipocytes in vivo.

## Figures and Tables

**Table 1:** Primer sequences of related gene for qPCR.

Gene	Primer Sequence (5′–3′)	Product Size (bp)	GeneBank No.
*INSR*	F: GTGTTGTGATTGGAAGCATTTAT	108	XM_021083940.1
	R: CATCGCTGGCACTGAGGTA		
*FABP4*	F: GCCAAACCCAACCTGATCAT	188	NM_001002817.1
	R: TCCCACTTCTGCACCTGTAC		
*PPARγ*	F: TTGCTGTGAAGTTCAACGCA	167	NM_214379.1
	R: GTGGTTCAACTTGAGCTGCA		
*IGF-1R*	F: CAACCTCCGGCCTTTTACTTT	134	NM_214172.1
	R: CAGGAATGTCATCTGCTCCTTCT		
*RPLP0*	F: TCCAGGCTTTAGGCATCACC	95	NM_001098598.1
	R: GGCTCCCACTTTGTCTCCAG		
*FASN*	F: GCTTGTCCTGGGAAGAGTGTA	114	NM_001099930.1
	R: AGGAACTCGGACATAGCGG		
*ACC*	F: CGCTTCATAATTGGTTCTGTG	147	NM_001114269.1
	R: GCTAGAAATCCCCAAGTCAGA		
*ATGL*	F: GCGAAAATGTCATCATAACC	175	NM_001098605.1
	R: ATGGTGCTCTTGAGTTCGT		
*HSL*	F: GCCCGAGACGAGATTAG	143	NM_214315.2
	R: ATGAAGGGATTCTTGACG		
*CPT-1α*	F: AACCTTCTGGCGGACGACG	213	NM_001129805.1
	R: GCAGGAACGCACGGTCTCA		
*CPT-1β*	F:ACTGTCTGGGCAAACCAAAC	176	NM_001007191.1
	R: CTTCTTGATGAGGCCTTTGC		
*PPARα*	F:CAGCCTCCAGCCCCTCGTC	382	NM_001044526.1
	R: GCGGTCTCGGCATCTTCTAGG		
*PGC-1α*	F: CTGTGGATGAAGACGGATTG	92	NM_213963.2
	R: GTCAGGCATGGAGGAAGGA		
*Cidea*	F: TTCCGAGTTTCCAACCACAA	97	NM_001112696
	R: CGATAACCAGGGCATCCAG		
*PRDM16*	F: CCACAAGTCCTACACGCAGTTCTC	121	XM_021095209.1
	R: GTTGAGGGACGAGGTAGTGCTGA		
*TFAM*	F:GGTCCATCACAGGTAAAGCTGAA	167	NM_001130211.1
	R:ATAAGATCGTTTCGCCCAACTTC		
*NRF-1*	F: CAACAGGAAAGAAACGGAAAC	157	XM_021078993.1
	R: GAGGGTGAGATACAAAGGACAAT		

## Abbreviations

INSR	Insulin receptor
IGF-1R	Insulin-like growth factor 1 receptor
MCM	Muscle conditional cultured medium
IMF	Intramuscular fat
DMEM	Dulbecco’s Modified Eagle’s medium
FBS	Fetal bovine serum
SDS-PAGE	Sodium dodecyl sulphate-polyacrylamide gel electrophoresis
PVDF	Polyvinylidene fluoride membranes
mtDNA	Mitochondrial DNA
COX2	Cytochrome c oxidase subunit II
RPLP0	Ribosomal protein lateral stalk subunit P0
PI	Propidium iodine
NRF-1	Nuclear respiratory factor-1
TFAM	Mitochondrial transcription factor A
WAT	White adipose tissue
BAT	Brown adipose tissue
